# Efficacy and safety of berberine for premature ventricular contractions: a meta-analysis and systematic review of randomized controlled trials

**DOI:** 10.1080/13880209.2023.2248167

**Published:** 2023-10-19

**Authors:** Meng Qiao, Chao Lei, Chaoren Tan, Cuncun Lu, Zijia Chen, Qiang Zhang, Zhifei Wang

**Affiliations:** a Institute of Basic Research in Clinical Medicine, China Academy of Chinese Medical Sciences, Beijing, PR China; b Institute of Acupuncture and Moxibustion, China Academy of Chinese Medical Science, Beijing, PR China

**Keywords:** *Coptis chinensis*, traditional Chinese medicine, complementary alternative medicine, antiarrhythmic drugs

## Abstract

**Context:**

Berberine is a potential drug that can effectively treat cardiovascular diseases, including premature ventricular contractions (PVCs).

**Objective:**

This study was conducted to assess the efficacy and safety of berberine for PVCs.

**Methods:**

The literature was searched using PubMed, Cochrane Library, Embase, China National Knowledge Infrastructure (CNKI), China Science and Technology Journal Database (VIP), Wanfang, and Chinese Biomedical Literature Database (CBM) for randomized controlled trials (RCTs) from inception to October 1, 2022. The risk of bias was assessed using the Revised Cochrane risk-of-bias tool for randomized trials, and the Grading of Recommendations Assessment, Development, and Evaluation (GRADE) system was adopted to assess the quality of evidence.

**Results:**

Ten RCTs with 896 participants were included in the meta-analysis. The results showed that compared to antiarrhythmic drugs (AD), berberine (BE) combined with AD had a higher effective rate (RR = 1.26; 95% CI:1.12, 1.42; *p* = 0.0001) with no significant incidence of adverse reactions (RR = 0.93; 95% CI:0.33, 2.57; *p* = 0.88), and BE alone had no significant difference in effective rate (RR = 0.91; 95% CI:0.77, 1.07; *p* = 0.23), and a lower incidence of adverse reactions (RR = 0.38; 95% CI:0.15, 0.97; *p* = 0.04) and recurrence rate (RR = 0.40; 95% CI:0.18, 0.88; *p* = 0.02).

**Conclusions:**

The results suggest that BE is an effective and safe adjunctive method for PVCs. In addition, BE is recommended for patients with PVCs who had severe adverse reactions after administrating AD as an alternative therapy.

## Introduction

As one of the most common types of cardiac arrhythmias, premature ventricular contractions (PVCs) are usually found in electrocardiogram examinations, with a prevalence of 1-4% in the general population, which increases with age, use of stimulants, hormonal changes, and presence of stress, insomnia, and fundamental diseases including cardiac diseases (Al-Khatib et al. 2018; Gorenek et al. 2020; Marcus 2020; Klewer et al. 2022). In general, patients with PVCs have no obvious symptoms, while some may have palpitations, chest tightness, dizziness, near-syncope, dyspnea, chest pain, fatigue, etc. (Gorenek et al. 2020; Marcus 2020). For patients with no or mild symptoms, a low PVC burden, and normal ventricular function, simple reassurance may be best, and for those with PVCs associated with symptoms or a reduced left ventricular ejection fraction, either medical treatments such as β-blockers, non-dihydropyridine calcium channel blockers, or catheter ablation are considered first-line therapies (Marcus 2020; Cao et al. 2022; Klewer et al. 2022). On the one hand, antiarrhythmic drugs (AD) have limited effect on normalizing the PVCs and maintaining sinus rhythm and are prone to cause severe side effects, while catheter ablation is costly and worked only for certain patients; on the other hand, a lot of evidence has demonstrated that traditional Chinese medicine is effective for PVCs (Zhang et al. 2021).

Berberine (BE) is an isoquinoline alkaloid derived from the roots, rhizomes, and stem bodies of plants of the Berberidaceae and Ranunculaceae families, and is one of the main bioactive components of *Coptis chinensis* Franch (Rhizoma Coptidis, Coptis, Ranunculaceae), a traditional Chinese medicine. First recorded in Shen Nong’s Herbal Classic, *Coptis chinensis* is widely used in Asian countries and shows efficacy and safety for a variety of diseases, including inflammatory and metabolic diseases, etc. (Ai et al. 2021; An et al. 2022). According to pharmacological studies, BE had a cardiovascular protective effect on various cardiovascular diseases (CVDs), including arrhythmia, atherosclerosis, hypertension, etc. (Cai et al. 2021; Lin et al. 2022). Besides, BE also has positive effects on lowering blood lipids and blood glucose, and controlling body weight and blood pressure (Zamani et al. 2022).

In recent years, the growing focus has been placed on BE for treating CVDs, and BE is a potential drug that could effectively treat cardiovascular diseases, including PVCs (Yang and Tong 2016; Chen et al. 2022). Therefore, we conducted this meta-analysis to assess the efficacy and safety of BE for PVCs and adopted the Grading of Recommendations Assessment, Development, and Evaluation (GRADE) system to assess the quality of evidence.

## Methods

This study was conducted as part of a systematic review and has been registered with the PROSPERO (International Prospective Register of Systematic Reviews) (registration number: CRD42023388337) (Qiao et al. 2023). The protocol followed the Preferred Reporting Items for Systematic Reviews and Meta-Analyses (PRISMA) guidelines (Page et al. 2021) and the Cochrane Handbook for Systematic Reviews of Interventions (Higgins and Thomas 2021).

### Inclusion and exclusion criteria

The inclusion criteria were as follows: (1) patients diagnosed with PVCs according to clinical diagnostic criteria (Society of Cardiac Pacing and Electrophysiology and Chinese Society of Biomedical Engineering 2003; Zipes et al. 2006; Cao et al. 2020). (2) intervention: BE or BE combined with AD. (3) comparison: AD. (4) outcomes: effective rate (as defined in each included study), incidence of adverse reaction (IAR), onset time (the period from the first day of intervention to the day reached the effective rate), recurrence rate, and electrocardiograph. (5) study design: RCTs.

Studies were excluded for the following reasons: (1) duplicate publications. (2) no relevant data or incomplete statistics for the effect size calculation. (3) case series, case reports, reviews, animal experiments, and pharmacological research. (4) the full text is not available. (5) original studies with suspected data errors.

### Search strategy

The literature was searched using three English electronic databases of PubMed, Embase, Cochrane Library, and four Chinese databases: CNKI, VIP, Wanfang, and CBM from inception to October 1, 2022. Medical subject heading (MeSH) terms and text words were used as follows: (‘Berberine’ OR ‘xiaobojian’ OR ‘huangliansu’) AND (‘premature ventricular contraction’ OR ‘ventricular premature contraction’). The language was limited to either English or Chinese.

### Study selection and data extraction

After removing duplications in Endnote X8, all titles and abstracts were screened according to eligibility criteria by two reviewers (Meng Qiao and Chao Lei) independently, and any discrepancy was resolved by inviting a third reviewer (Cuncun Lu) to discuss and decide. Next, the full texts were read. Finally, eligible studies were selected, and with a standardized form, two investigators (Meng Qiao and Chao Lei) independently extracted the following data: (1) basic information: author, publication year, sample size, sex, age, and baseline characteristics; (2) intervention measures, duration, and outcomes of the intervention and comparison groups. The authors of the included studies were contacted if relevant information was not available.

### Assessment of risk of bias

Two reviewers (Meng Qiao and Chaoren Tan) independently assessed the risk of bias of the included studies using the Revised Cochrane risk-of-bias tool for randomized trials (RoB 2), which contains five domains: bias arising from the randomization process, bias due to deviations from intended interventions, bias due to missing outcome data, bias in measurement of the outcome, bias in selection of the reported result, and an overall risk of bias (Sterne et al. 2019). The included clinical trials were classified as low risk (low risk of bias for all five domains), some concerns (some concerns of bias for at least one domain but without high risk), or high risk (high risk of bias for at least one domain) according to the five domains. Any discrepancies were resolved by inviting a third reviewer (Cuncun Lu) to discuss and decide.

### Statistical analysis

Review Manager 5.3 was used for statistical analysis. Risk ratio (RR) was used as the effect indicator of dichotomous variables, and the effect size was represented by the 95% confidence interval (CI). References will be made to the Cochrane Handbook recommendations for design, data synthesis, and analysis strategies. The heterogeneity of the studies was evaluated by I square (I^2^) and P value (P). When *p* > 0.1 and I^2^ ≤ 50%, the fixed-effect model was adopted. If *p* ≤ 0.1 or I^2^ > 50%, it was considered to indicate substantial heterogeneity, and after exploring heterogeneity and removing obvious heterogeneity, a random-effects model was applied. Statistical significance was set at *p* ≤ 0.05. Statistical analyses were conducted using Review Manager version 5.3.

### Sensitive analysis and publication bias

A sensitivity analysis of the primary outcomes (effective rate and IAR) was conducted. Publication bias of primary outcomes was detected using funnel plots and the Luis Furuya-Kanamori (LFK) index. If an LFK index > 1 or < −1 and a visual inspection of the funnel plot showed asymmetry, publication bias was considered. Sensitivity analysis and publication bias analysis were conducted using Stata 12.0.

### Assessment of evidence quality

Two reviewers (Meng Qiao and Chaoren Tan) independently assessed the overall quality of evidence of the effective rate, IAR, and recurrence rate using the GRADE system (Brozek et al. 2009). According to five domains: limitation of design, inconsistency, indirectness, imprecision, and publication bias, the quality of the evidence was rated one of the four levels: high quality, moderate quality, low quality, and very low quality.

## Results

### Studies included

**Figure 1.**
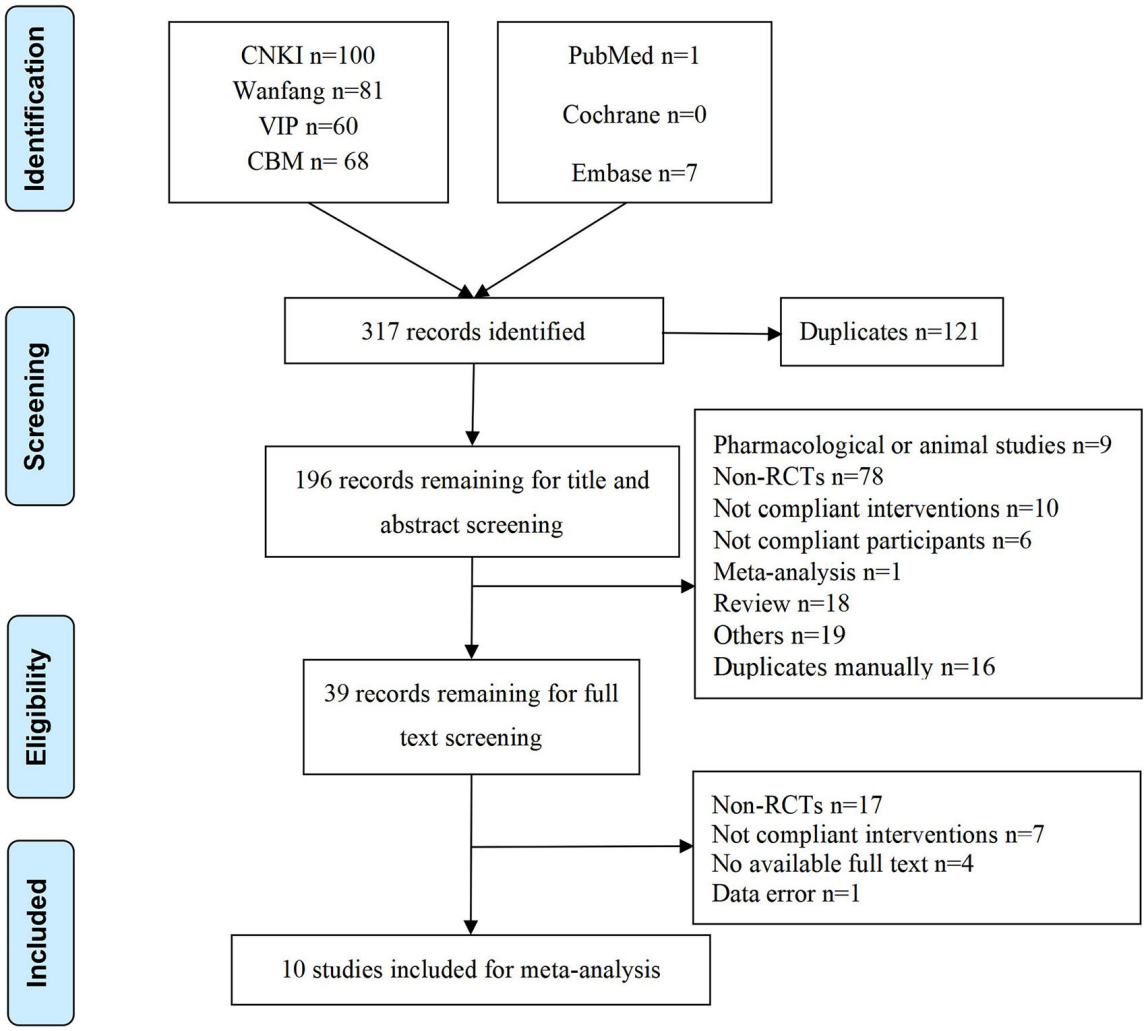
Flowchart of literature screening. CNKI, China National Knowledge Infrastructure; VIP, China Science and Technology Journal Database; CBM, Chinese Biomedical Literature Database.

A total of 317 records were identified from seven databases. After duplication in Endnote X8, 196 records remained for screening titles and abstracts. Thus, 39 records remained for full-text screening. Finally, 10 articles were included in the meta-analysis. The literature retrieval and selection processes are shown in Figure 1.

### Study characteristics

The final studies (Li and Li 1993; Shen and Liu 1994; Diao and Zou 1999; Zhao et al. 2000; Wang et al. 2003; Fan et al. 2004; Hu and Hu 2006; Shao et al. 2009; Rao and Yu 2010; Su et al. 2010) including 896 participants (Intervention/Comparison:469/427), were conducted in China from 1993 to 2010. The sample size varied from 35 to 263. Seven studies (Diao and Zou 1999; Zhao et al. 2000; Wang et al. 2003; Hu and Hu 2006; Shao et al. 2009; Rao and Yu 2010; Su et al. 2010) reported no significant baseline characteristics between intervention and comparison groups, while the remaining 3 (Li and Li 1993; Shen and Liu 1994; Fan et al. 2004) did not report baseline characteristics. Participants included both children and adults. The duration of the intervention varied from 7 to 30 days. All drugs were administered orally. The dose of BE each time was 0.3-0.6 g and less than 0.4 g for children, three or four times a day. Seven studies (Shen and Liu 1994; Diao and Zou 1999; Zhao et al. 2000; Wang et al. 2003; Hu and Hu 2006; Rao and Yu 2010; Su et al. 2010) compared BE combined with AD, and three studies (Li and Li 1993; Fan et al. 2004; Shao et al. 2009) compared BE with AD, in which AD included mexiletine, propafenone, and amiodarone. All studies (Li and Li 1993; Shen and Liu 1994; Diao and Zou 1999; Zhao et al. 2000; Wang et al. 2003; Fan et al. 2004; Hu and Hu 2006; Shao et al. 2009; Rao and Yu 2010; Su et al. 2010) have reported effective rate, 9 (Li and Li 1993; Diao and Zou 1999; Zhao et al. 2000; Wang et al. 2003; Fan et al. 2004; Hu and Hu 2006; Shao et al. 2009; Rao and Yu 2010; Su et al. 2010) reported IAR, 4 reported onset time (Li and Li 1993; Diao and Zou 1999; Fan et al. 2004; Hu and Hu 2006) and electrocardiograph (Li and Li 1993; Wang et al. 2003; Fan et al. 2004; Rao and Yu 2010) and 2 (Zhao et al. 2000; Hu and Hu 2006) reported recurrence rate. The details are listed in Table 1.

**Table 1.** Detailed information of the included studies.

Author and year	Type of PVC	Comparable baseline characteristics	Sample size		Age (year)		Intervention method		Administration method	Duration	Outcome measures
			I	C	I	C	I	C			
Li and Li 1993	Refractory PVC	--	30	20	38 ± 11.2		Berberine (0.4 g/time, 3 times/d) + Mexiletine (0.1 g/time, 3 times/d)	Mexiletine (0.15 g/time, 3 times/d)	P.O	--	①②④⑤
Shen and Liu 1994	Frequent PVC	--	20	15		23-64	18-60	Berberine (0.4-0.6 g/time, 4 times/d)	Mexiletine (0.1 g/time, 3 times/d)	P.O	3W	①
Diao and Zou 1999	Elderly with frequent PVC	Y	64	64	66 ± 5		Berberine (0.4 g/time, 3 times/d)	Propafenone (0.15 g/time, 3 times/d)	P.O	4W	①②④	
Zhao et al. 2000	Simple frequent PVC	Y	30	30	18-40		Berberine (0.4 g/time, 4 times/d)	Propafenone (0.2 g/time, 4 times/d)	P.O	2W	①②③	
Wang et al. 2003	Elderly with frequent PVC	Y	39	32	57 ± 17	52 ± 15	Berberine (0.5 g/time, 4 times/d)	Mexiletine (0.1 g/time, 4 times/d)	P.O	1W	①②⑤	
Fan et al. 2004	PVC	--	142	121	15-69	15-68	Berberine (0.3 g/time, 3 times/d) + Propafenone (0.1 g/time, 3 times/d)	Propafenone (0.1 g/time, 3 times/d)	P.O	4W	①②④⑤	
Hu and Hu 2006	Functional PVC	Y	42	43	18-54		Berberine (0.4 g/time, 3 times/d)	Amiodarone (Day 1-3: 0.2 g/time, 3 times/day; After: 0.2 g/time, once/d)	P.O	1M	①②③④	
Shao et al. 2009	PVC	Y	36	36	45.1 ± 6.4	44.8 ± 5.2	Berberine (0.3 g/time, 3 times/d)+ Propafenone (0.1 g/time, 3 times/d)	Propafenone (0.1 g/time, 3 times/d)	P.O	30D	①②	
Rao and Yu 2010	Children with PVC	Y	36	36	9.5 ± 3	9.8 ± 2.5	Berberine (15-20 mg/kg·d, Max ≤ 0.4g/d, 3 times/d)	Amiodarone (2.5-5 mg/(kg·time), Day 1-3: 3 times/day; After: twice/day)	P.O	2-4W	①②⑤	
Su et al. 2010	Functional PVC	Y	30	30	19-56	18-58	Berberine (0.4 g/time, 3 times/d)	Propafenone (0.1 g/time, 3 times/d)	P.O	1W	①②	

Abbreviations: VPBs, premature ventricular contraction; Y, yes; I, intervention; C, control; d, day; P.O, per os; W, week.  ①, Effective rate; ②, Incidence of adverse reaction; ③, Recurrence rate; ④, Onset time; ⑤, Electrocardiogram.

### Risk of bias

**Figure 2.**
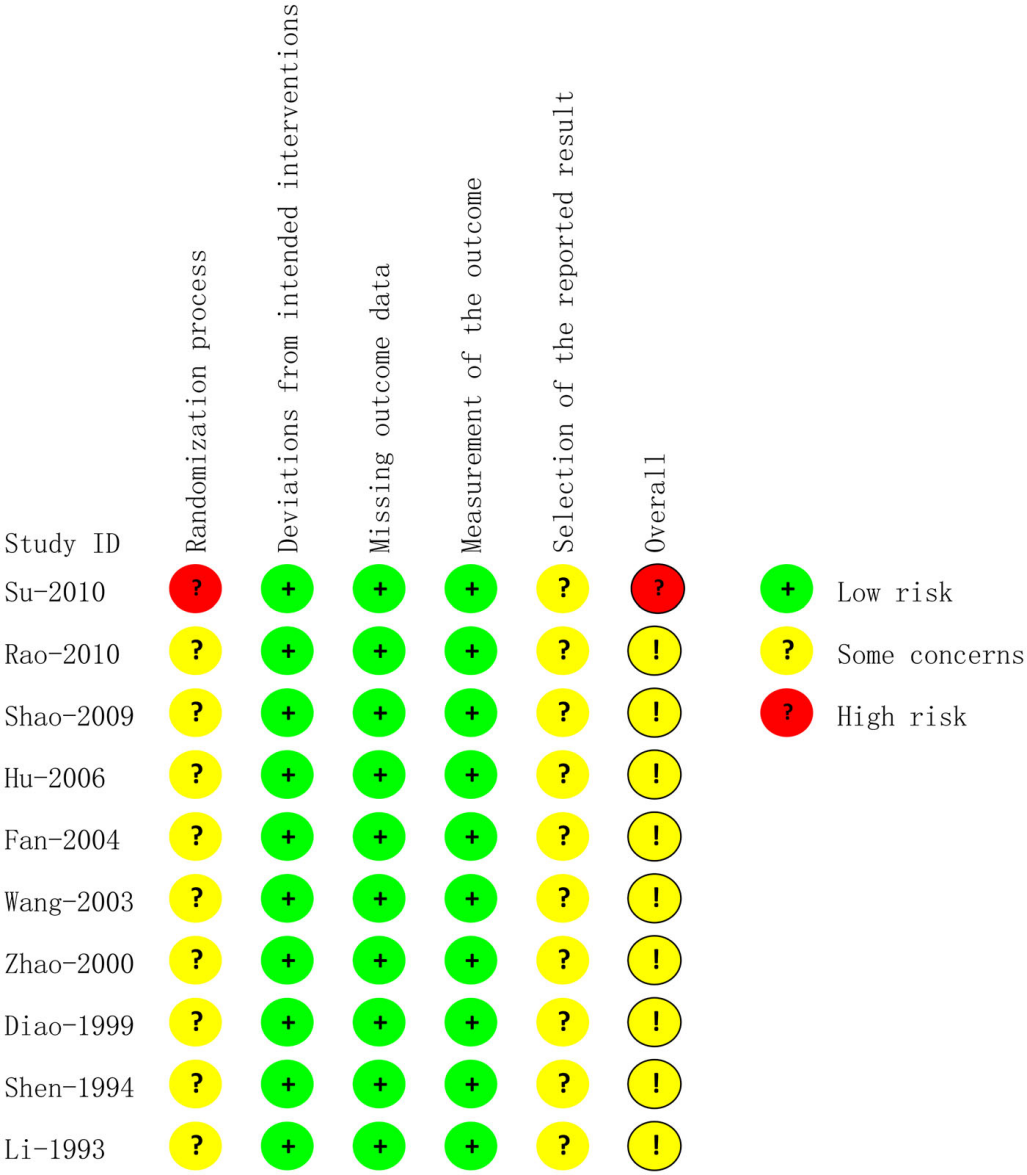
Risk of bias assessment for included studies.

In the randomization process domain, no study reported concealment of randomization, and one study (Su et al. 2010) rated the high risk as it allocated participants to intervention or comparison groups according to the order of visit, and the rest were assessed for some concerns. All studies were assessed as low risk in the domain of deviations from intended interventions because we considered that all the included studies adopted ITT to analyze the data. No data were missing, so we rated the missing outcome data as low risk. All studies were rated as low risk in the measurement of the outcome because they adopted an appropriate method to measure outcomes, which was the same between intervention groups. All studies had some concerns regarding the selection of the reported results, as they reported no description of the study proposal or registration. A summary of the risk of bias is shown in Figure 2.

### Primary outcome

#### Effective rate

**Figure 3.**
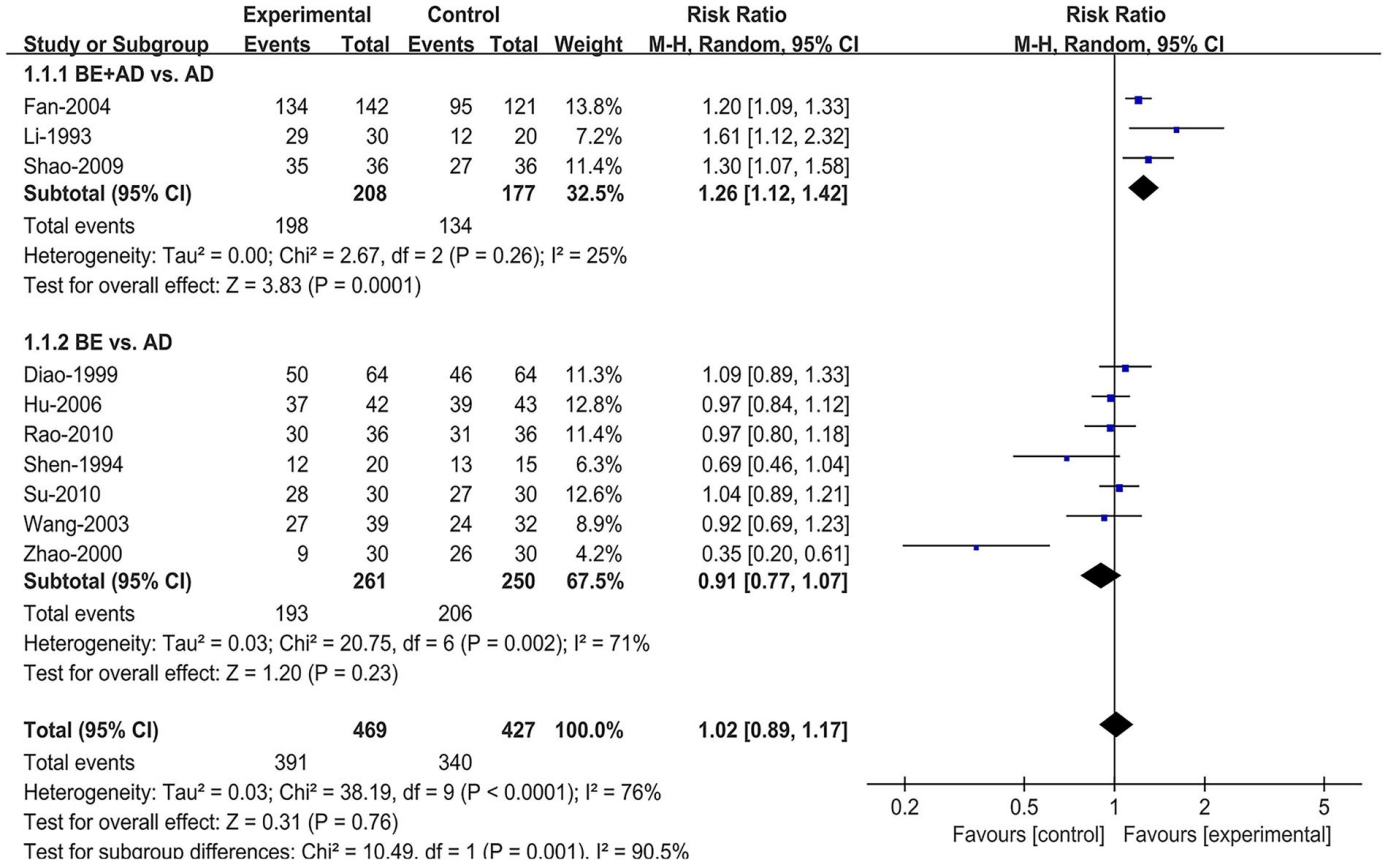
Forest plot comparing effective rate between berberine and control groups. AD: antiarrhythmic drugs; BE: berberine; CI: confidence interval.

All ten studies (Li and Li 1993; Shen and Liu 1994; Diao and Zou 1999; Zhao et al. 2000; Wang et al. 2003; Fan et al. 2004; Hu and Hu 2006; Shao et al. 2009; Rao and Yu 2010; Su et al. 2010) reported the effective rate. Due to the high heterogeneity (I^2^ = 76%), we conducted a subgroup analysis based on intervention type, adopting a random effect model for synthesis. Three (Li and Li 1993; Fan et al. 2004; Shao et al. 2009) of them compared BE combined with AD to AD alone and the remaining seven (Shen and Liu 1994; Diao and Zou 1999; Zhao et al. 2000; Wang et al. 2003; Hu and Hu 2006; Rao and Yu 2010; Su et al. 2010) compared BE directly to AD. The results of the meta-analysis showed that compared to AD, BE combined with AD had a higher effective rate (RR = 1.26; 95% CI:1.12, 1.42; *p* = 0.0001), and BE alone had no significant difference in the effective rate (RR = 0.91; 95% CI:0.77, 1.07; *p* = 0.23) (Figure 3).

#### Incidence of adverse reaction

**Figure 4.**
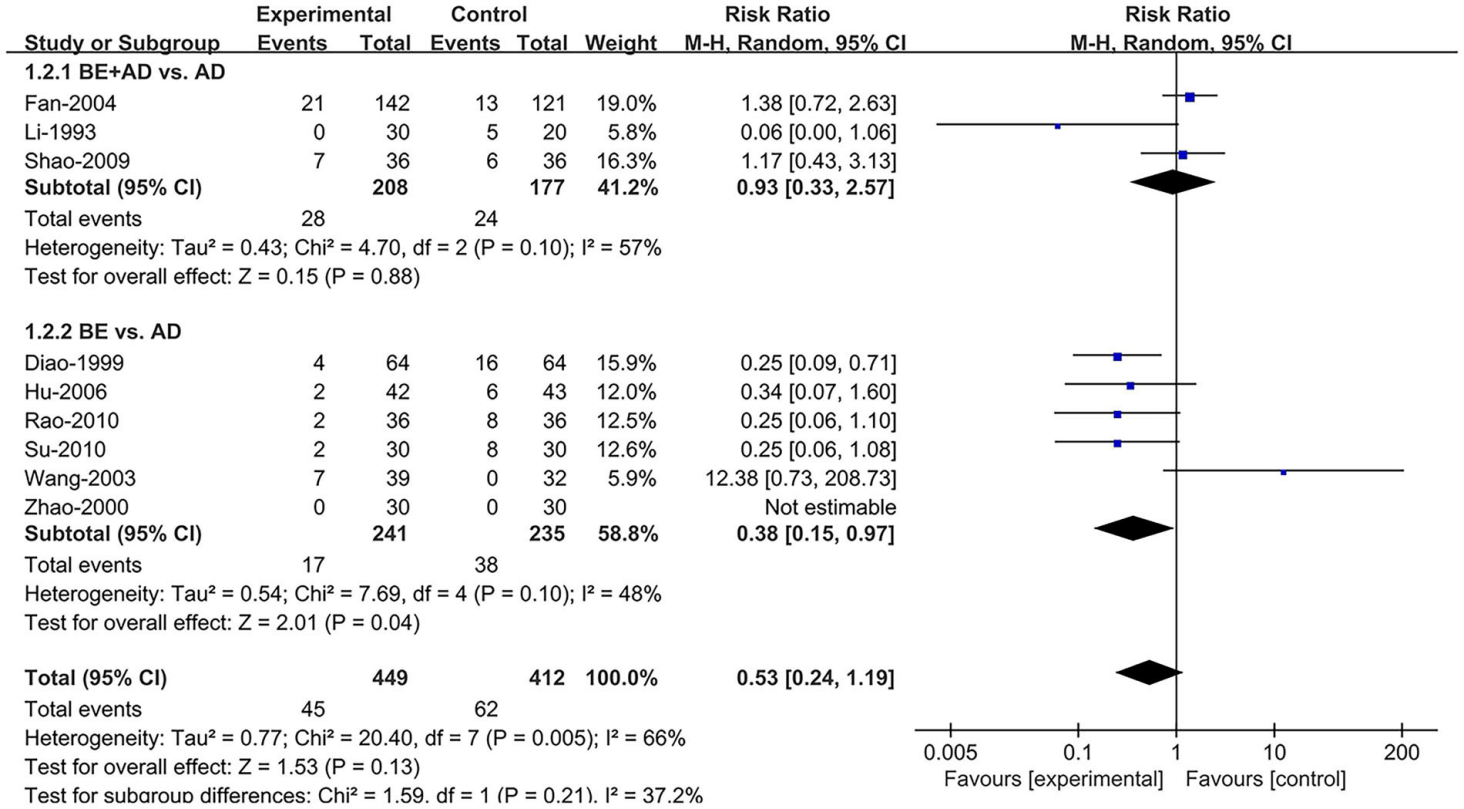
Forest plot comparing incidence of adverse reactions between berberine and control groups. AD: antiarrhythmic drugs; BE: berberine; CI: confidence interval.

Nine studies (Li and Li 1993; Diao and Zou 1999; Zhao et al. 2000; Wang et al. 2003; Fan et al. 2004; Hu and Hu 2006; Shao et al. 2009; Rao and Yu 2010; Su et al. 2010) reported the IAR. A random effects model was adopted, and subgroup analysis based on intervention type was conducted owing to heterogeneity (I^2^ = 66%). Compared to AD, BE combined with AD (RR = 0.93; 95% CI:0.33, 2.57; *p* = 0.88) had no significant difference in IAR, while BE (RR = 0.38; 95% CI:0.15, 0.97; *p* = 0.04) alone had a lower IAR (Figure 4).

### Secondary outcome

#### Recurrence rate

**Figure 5.**
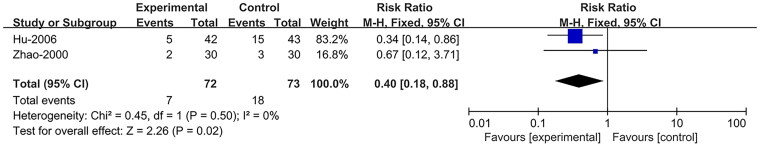
Forest plot comparing recurrence rate between berberine and control groups. AD: antiarrhythmic drugs; BE: berberine; CI: confidence interval.

Two studies (Zhao et al. 2000; Hu and Hu 2006) reported recurrence rate and compared BE with AD. The fixed effects model was adopted, and the results showed that the BE group had a lower recurrence rate than the AD group (RR = 0.40; 95% CI:0.18, 0.88; *p* = 0.02) (Figure 5).

#### Onset time

Four studies (Li and Li 1993; Wang et al. 2003; Fan et al. 2004; Rao and Yu 2010) reported onset time. A descriptive analysis was used to analyze the data because they were presented in different ways. Li and Li (1993) reported that the onset time of the intervention group was 2-12 days, with an average of 7 days, and that of the comparison group was 3-30 days, with an average of 18 days. Diao and Zou (1999) found the onset time was 8.3 ± 1.1 days and 7.9 ± 1.2 days for intervention and comparison groups respectively, with no significant difference. Fan et al. (2004) reported a relatively shorter onset time in the intervention group (3-21 days) than that of the comparison group (7-30 days). Hu and Hu (2006) reported that the onset time for the intervention group were 4-7 days and 6-8 days for the comparison group respectively. Overall, the intervention group had shorter onset time.

#### Electrocardiograph

Four studies (Li and Li 1993; Wang et al. 2003; Fan et al. 2004; Rao and Yu 2010) reported electrocardiograph, 2 (Li and Li 1993; Fan et al. 2004) compared BE combined with AD to AD, and the other 2 (Wang et al. 2003; Rao and Yu 2010) compared BE to AD. Descriptive statistics were used to analyze the data because they could not be pooled. Li and Li (1993) reported that after intervention, the PR and QT intervals and the QRS period did not change. Wang et al. (2003) and Fan et al. (2004) reported that after intervention, both the intervention and comparison groups showed no change in PR, PRS, and QT interval. Rao and Yu (2010) reported that compared with amiodarone in the control group, BE had no obvious proarrhythmic and negative inotropic effects and did not affect ventricular conduction, aggravate heart failure, or prolong Q-T interval. In conclusion, both the intervention and comparison groups showed no significant change in electrocardiograph after intervention.

### Sensitivity analysis and publication bias

**Figure 6.**
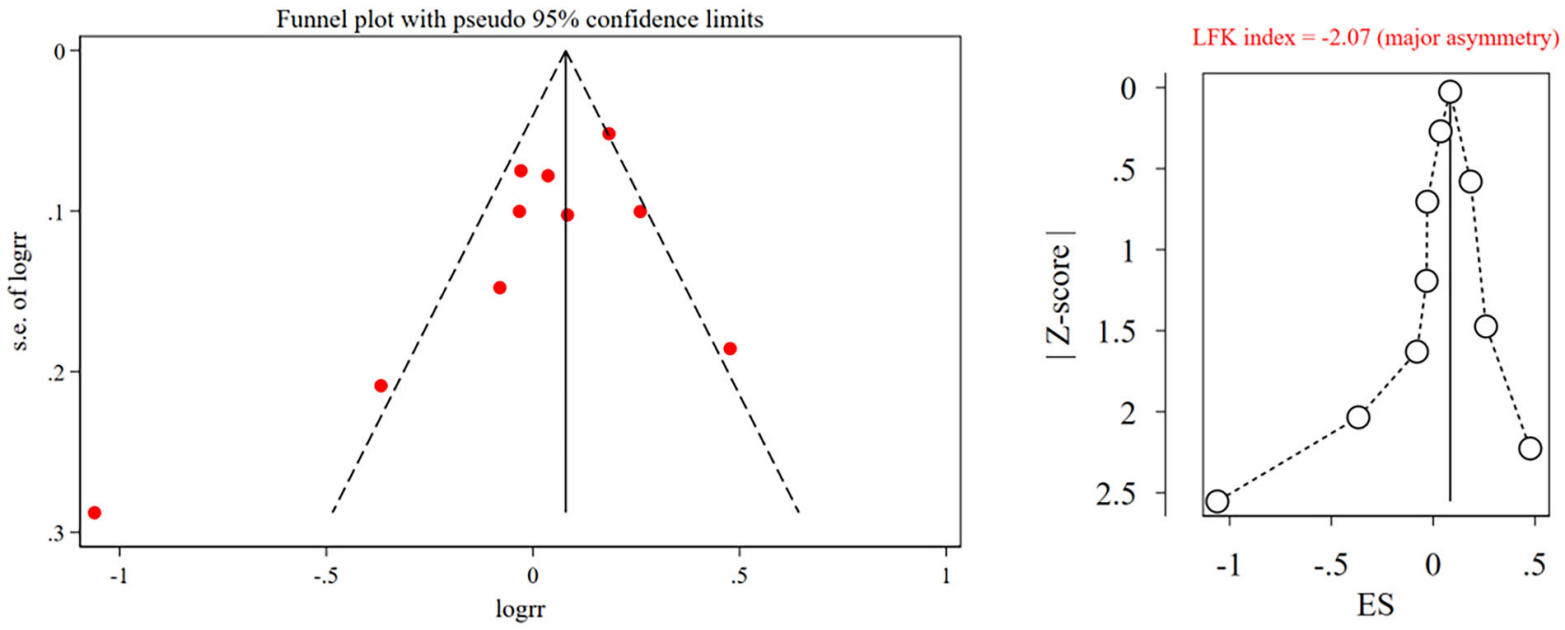
Funnel plot and LFK index for effective rate. RR, risk ratio; ES, effect size; LFK, Luis Furuya-Kanamori.

**Figure 7.**
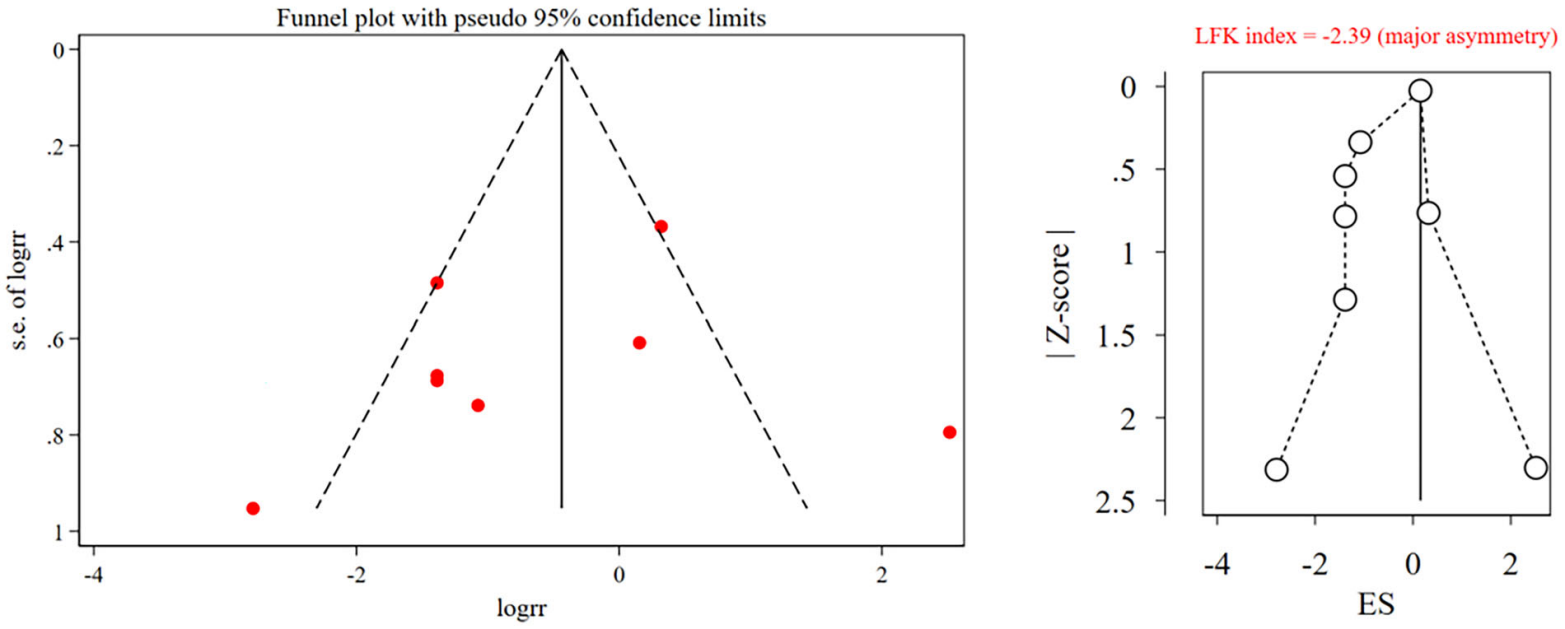
Funnel plot and LFK index for incidence of adverse reactions. RR, risk ratio; ES, effect size; LFK, Luis Furuya-Kanamori.

The results of the sensitivity analysis showed that the pooled results of effective rate were stable, while that of IAR were unstable because after excluding Wang et al. (2003), the pooled results changed significantly (Tables 2 and 3). The funnel plot of both effective rate and IAR showed asymmetry and an LFK index <0.1 in both outcomes; therefore, we considered that there was publication bias (Figures 6 and 7).

**Table 2.** Sensitivity analysis of effective rate.

Study omitted	Estimate	[95% Conf. Interval]	
Li and Li 1993	0.9880937	0.8608739	1.134114
Shen and Liu 1994	1.0511261	0.91920905	1.2019748
Diao and Zou 1999	1.0086187	0.86545011	1.1754712
Zhao et al. 2000	1.0748688	0.96665653	1.1951948
Wang et al. 2003	1.0305038	0.89185865	1.1907021
Fan et al. 2004	0.99044955	0.8468745	1.1583656
Hu and Hu 2006	1.0229185	0.87527253	1.1954702
Shao et al. 2009	0.98959366	0.85501489	1.145355
Rao and Yu 2010	1.0250245	0.88168004	1.191674
Su et al. 2010	1.0121326	0.86395628	1.1857224
Combined	1.0215328	0.89192993	1.1699678

**Table 3.** Sensitivity analysis of incidence of adverse reaction.

Study omitted	Estimate	[95% Conf. Interval]	
Li and Li 1993	0.61077421	0.27636054	1.3498495
Diao and Zou 1999	0.61943099	0.2619277	1.464888
Zhao et al. 2000	0.534898	0.24016813	1.1913149
Wang et al. 2003	0.4500928	0.20707728	0.97829913
Fan et al. 2004	0.42787718	0.18132258	1.009686
Hu and Hu 2006	0.56618378	0.23251874	1.3786591
Shao et al. 2009	0.45673449	0.17645156	1.1822304
Rao and Yu 2010	0.59512734	0.24949441	1.4195771
Su et al. 2010	0.59574562	0.2496793	1.4214749
Combined	0.534898	0.24016813	1.1913149

### Assessment of evidence quality

GRADE profiler 3.6 was used to draw a summary of the quality of evidence. Low quality of evidence showed that compared with AD, BE combined with AD had a higher effective rate, and BE alone had similar effective rate to AD with significantly lower IAR and recurrence rate than those of AD. Very low-quality evidence showed that BE combined with AD had similar IAR to AD. They were downgraded mainly because of a lack of randomization, concealment of randomization and blinding, high heterogeneity, and wide confidence intervals (Table 4).

**Table 4.** GRADE certainty assessments and summary of findings for outcomes.

Quality assessment						No of patients		Effect		Quality	Importance
No of studies	Risk of bias	Inconsistency	Indirectness	Imprecision	Other considerations	Berberine	Control	Relative (95Cl%)	Absolute		
Effective rate – BE + AD vs. AD											
3 RCTs	serious^1^	no serious^2^	no serious	serious^3^	none^4^	198/208(95.2%)	134/177 (75.7%)	RR 1.26 (1.12,1.42)	197 more per 1000 (from 91 more to 318 more)	**Low**	**Critical**
							75%		195 more per 1000 (from 90 more to 315 more)		
Effective rate – BE vs. AD											
7 RCTs	serious^1^	serious^5^	no serious	no serious^6^	none^4^	193/261 (73.9%)	206/250 (82.4%)	RR 0.91 (0.77, 1.07)	74 fewer per 1000 (from 190 fewer to 58 more)	**Low**	**Critical**
							86.7%		78 fewer per 1000 (from 99 fewer to 61 more)		
Incidence of adverse reactions – BE + AD vs. AD											
3 RCTs	serious^1^	serious^7^	no serious	very serious^8^	none^4^	28/208 (13.5%)	24/177 (13.6%)	RR 0.93 (0.33, 2.57)	9 fewer per 1000 (from 91 fewer to 213 more)	**Very Low**	**Critical**
							16.7%		12 fewer per 1000 (from 112 fewer to 262 more)		
Incidence of adverse reactions – BE vs. AD											
6 RCTs	serious^1^	no serious^9^	no serious	serious^3^	none^4^	17/241 (7.1%)	38/235 (16.2%)	RR 0.38 (0.15,0.97)	100 fewer per 1000 (from 5 fewer to 137 more)	**Low**	**Critical**
							18.1%		112 fewer per 1000 (from 5 fewer to 154 more)		
Recurrence rate – BE vs. AD											
2 RCTs	serious^1^	no serious^10^	no serious	serious^3^	none^4^	7/72 (9.7%)	18/73 (24.7%)	RR 0.40 (0.18,0.88)	148 more per 1000 (from 30 more to 202 more)	**Low**	**Important**
							22.4%		134 more per 1000 (from 27 more to 184 more)		

GRADE, Grading of Recommendations Assessment, Development, and Evaluationc.

^1^Most studies were ranked unclear risk based on Cochrane risk of bias tool, primarily due to lack of randomzation, concealment of randomization and blinding. ^2^I-square = 25% ^3^Confidence interval include appreciable harm and benefit. ^4^Most studies include participants over 30 pairs and there are no signs for studies sponsored by industry. ^5^I-square = 71%. ^6^Confidence interval do not include appreciable harm and benefit, and the total events > 300. ^7^I-square = 57%. ^8^Confidence interval include null effect and include appreciable harm and benefit.

^9^I-square = 48%.

^10^I-square = 0%.

## Discussion

We conducted a comprehensive search of seven databases to provide the latest evidence that BE is an effective and safe therapy for treating PVCs. About the effective rate, BE combined with AD was significantly better than AD with no significant IAR, which mainly included liver damage and digestive system symptoms, and the liver damage disappeared after liver-protection treatment. In addition, the AD group also experienced adverse reactions, including bradycardia. BE alone had a similar effective rate to AD, with a lower IAR and recurrence rate. The main IAR for BE was digestive system symptoms, which were mild and disappeared after discontinuation of medication or spontaneously remitted. Besides digestive system symptoms, the IAR for AD also included cardiovascular symptoms such as bradycardia, atrioventricular block, hypotension, and dizziness, and some patients were forced to stop the intervention due to intolerance. The BE group had a shorter onset time and little effect on electrocardiograph than the comparison group.

Guo (2015) found that, compared with AD, BE had a higher effective rate of PVCs with lower IAR. But subgroup analysis based on intervention type showed that in comparison to AD, BE had a similar effective rate while BE combined with AD had a higher effective rate than AD. Therefore, we recommend that BE should be combined with AD for PVCs. The side effects of AD in digestive tract varied, including esophagitis, esophageal ulcer, acute pancreatitis, and constipation. Different types of AD have different side effects, such as hepatotoxicity, pulmonary toxicity, thyroid dysfunction, hypersensitivity, etc. (Amjad et al. 2017). BE is mainly absorbed in the intestinal tract, and a high dose of BE could result in gastrointestinal symptoms which were mild and tolerable (Cai et al. 2021). BE combined with AD could alleviate, to some extent, the side effects of the cardiovascular system caused by AD.

The results showed that there was a publication bias in the effective rate and IAR. The funnel plot and LFK index were used to evaluate the publication bias. The funnel plot was intuitive but subjective, whereas the LFK index (Furuya-Kanamori et al. 2018) avoided subjective judgment and was the best in terms of statistical performance and bias detection rate compared with Egger’s test and Begg’s test. This combination of objective and subjective methods solidified the results of the publication bias. One possible reason for this bias is that all ten included studies were conducted and published in China. Apart from this, none of the included studies reported funding sources, conflicts of interest, or research proposals, which may also result in publication bias. By conducting a sensitivity analysis, excluding the included studies one by one, the results of the effective rate were stable, while those of IAR were not. Subgroup analysis was conducted, and the results showed that after excluding the Wang et al. (2003), the change in the effect size of the subgroup belonging to this study was small. Therefore, subgroup analysis is meaningful.

This study adopted the GRADE system to assess the quality of evidence for three outcomes: effective rate, IAR, and recurrence rate. Due to the low to very low quality of evidence, this evidence should be interpreted with caution when making clinical decisions, and the overall profile of the patient should be taken into account. Of note, we considered downgrading the quality of evidence when the sample size was less than 30 pairs and the study was funded by manufacturers, which was different from assessing the publication bias using funnel plot and LFK index.

BE may exert an antiarrhythmic effect by controlling oxidative stress and reducing myocardial damage. BE blocks K^+^ channels, stimulates Na^+^-Ca^2+^ exchanger, increases coronary blood flow to increase myocardial contractility and cardiac output, reduces the prolongation of monophasic action potential repolarization, and reduces the occurrence of PVCs (Feng et al. 2019; Cai et al. 2021; An et al. 2022). In addition to improving PVCs, BE also has high clinical prospects for improving the long-term prognosis of heart disease, as confirmed by its protective effects on the vascular endothelium and improving cardiac function (Cai et al. 2021). However, BE has poor bioavailability due to the first-pass effect in the intestinal lumen, and all included studies administered BE orally, which might have influenced its efficacy (Wang et al. 2017; Xu et al. 2019; Habtemariam 2020).

This research was registered in PROSPERO and reported following the Meta-Analyses (PRISMA) guidelines. This study had some limitations. First, all included studies were single-center studies conducted in China, and the sample size for most of them was small, which may have resulted in publication bias. Second, the publication time of the included studies varied from 1993 to 2010, and inconsistencies were observed among them, including diagnostic criteria, doses, and outcomes, which might affect the authenticity of the results. Third, most studies lacked detailed descriptions of randomization, concealment of allocation, blinding of participants, and research and outcome assessors. Fourth, the quality of evidence was low to very low; therefore, it should be considered carefully when recommending evidence. Fifth, a relatively long follow-up is required to assess the efficacy of treatment for PVCs, while the longest duration of included studies was 30 days, which may have influenced our evaluation of the results.

## Conclusions

The results of this study suggest that BE is an effective and safe adjunctive method for PVCs. In addition, BE is recommended for patients with PVCs who had severe adverse reactions after administrating AD as an alternative therapy. However, owing to the low or very low quality of evidence, this evidence should be considered carefully and more rigorously designed RCTs are warranted to support our conclusions further.
